# Short and Long Term Investor Synchronization Caused by Decoupling

**DOI:** 10.1371/journal.pone.0050700

**Published:** 2012-12-07

**Authors:** Magda Roszczynska-Kurasinska, Andrzej Nowak, Daniel Kamieniarz, Sorin Solomon, Jørgen Vitting Andersen

**Affiliations:** 1 The Robert B. Zajonc Institute for Social Studies, University of Warsaw, Warsaw, Poland; 2 University of Social Science and Humanities, Warsaw, Poland; 3 Department of Psychology, Florida Atlantic University, Boca Raton, Florida, United States of America; 4 Racah Institute of Physics, The Hebrew University of Jerusalem, Jerusalem, Israel; 5 CNRS, Centre d'Economie de la Sorbonne, Université Paris 1 Panthéon-Sorbonne, Paris, France; Universidad Carlos III de Madrid, Spain

## Abstract

The dynamics of collective decision making is not yet well understood. Its practical relevance however can be of utmost importance, as experienced by people who lost their fortunes in turbulent moments of financial markets. In this paper we show how spontaneous collective “moods” or “biases” emerge dynamically among human participants playing a trading game in a simple model of the stock market. Applying theory and computer simulations to the experimental data generated by humans, we are able to predict the onset of such moments **before** they actually happen.

## Introduction

The existence of forecasting patterns in asset prices has, for obvious reasons, been a widely explored and discussed topic in the financial industry as well as academic literature. It should however be noted that the majority of the documented cases refer to **ex post** analysis, see [1]–[3]. In order to study the general problem of the dynamics of collective decision making and eventually predict the outcome of an aggregate decision making **before** a general “consensus” is reached, we suggest considering a very simple model of the stock market. As it will be shown, we provide a method for finding pockets of predictability in the decision making when humans are made to trade according to the setup of the model. Besides we offer a rationale of the humans' behavior that drives the majority of them to a temporary synchronization.

In financial markets the returns of asset prices are believed to be temporally independent, meaning that today's return does not predict in any way the sign of tomorrow's return. This is especially the case when it is assumed that investors' expectations are unbiased [4], [5]. In the 60 s and 70 s such assumption paved the way to the efficient market hypothesis, arguing that future returns cannot be predicted from past returns or any other market-based indicator [6]. However, findings of behavioral economics challenged this assumption showing that people tend to make systematic cognitive errors when forming expectations, as it can be seen in the case of representativeness or anchoring heuristics [7]–[10]. Other research also shows that people tend to create speculative price bubbles independently of the experimental setting [11]–[13]. This means that under some circumstances, the investors' expectations tend to become biased [14] and once they are biased – they become predictable.

Temporal loss of people's capability to adapt is especially common during a rapid change of a trend. If we apply this rule to investment decisions in financial markets, a sudden trend change can lead to a severe decrease of investors' performance and a subsequent evaporation of fortunes. During regular performance of the market, on the contrary, active, short-term investors react dynamically to incoming information [15], [16]. While making predictions about the future state of the market they often analyze past price changes, what is called technical analysis. Predictions based on technical analysis are usually sensitive to the next outcome of the market; a different recommendation will be given in case of a positive or negative change of price in the consecutive time step. However, under some specific market history it is possible that no matter what the next state of the market, the investor's strategy will recommend the same decision, i.e., buy or sell. In such situation, the decision is independent of what will happen next - it appears to be decoupled from the immediate market change. An investor is no longer influenced by the incoming information because all information will drive them to the same conclusion, making the market predictable. We call this cognitive mechanism “decoupling” and will give its precise definition below.

We hypothesize that when a majority of investors experience decoupling, the market dynamics changes dramatically. Investors become locked in their positions, and their decision heuristics are immune to disconfirming information. They become incapable of reacting to alarming signals what consolidates their synchronization, or even leads to the creation of bubbles or anti-bubbles (continuous increases or decreases of prices).

In the following we will show a method by which soft human decision heuristics can be formalized in terms of decision rules of agents in an agent-based simulation of the financial market called the $-Game ($G). The general idea is to have a framework in which one can study the dynamics of collective decision making, knowing the factors relevant for the decision making of an individual. The problem will be presented in a simple model of the stock market, but the questions posed belong to the general domain of collective decision making, where the aggregate choice feeds back on the formation of individual choices.

Within the simple agent-based model of the stock market, we run a Monte Carlo simulation and demonstrate that:

- First of all, decoupling can lead to biased price formation that may evolve into bubbles or anti-bubbles.
- Secondly, the detection of decoupling allows to detect biased price behavior before a bubble (or anti-bubble) appears.

In experiments with uniquely human participants, we show that in an analogous setup the subjects follow similar price dynamics as agents in simulations, which is indicated by the appearance of bubbles or anti-bubbles. Moreover, when we used data generated by humans as input for the artificial agents, we show that:

- Decoupling can explain the synchronization of human subjects.
- Ratio of decoupled strategies used by artificial agents can predict (with high probability) the creation of a speculative price bias by humans.

### The $-Game: a model of a financial market and a mathematical formulation of human decision heuristics

We have chosen the $G because, first of all, it is an extremely simple model of financial markets. Secondly, the rules of the $G facilitate the emergence of collective speculative biases – the market phenomena whose dynamics we want to explore and understand. In the following we therefore explain its rules in detail [17].

Specifically the $-Game is described by just three parameters (N, m, s):

- N - Number of agents (market participants)
- m - “Memory” used by the decision making of the agents. The parameter m represents the past number of days used by the agents in their decision making of whether to buy or sell an asset. Therefore, m is the length of the signal used in the decision making, see Table 1.
- s - Number of strategies held by the agents

**Table 1:** Decision table showing an example of a strategy that uses m = 2 recent time steps.

price history	action
00	+1
01	−1
10	+1
11	+1

In the $G, agents can either buy or sell one unit of stock at each time step; they are assumed to have an unlimited amount of money and stock. The decision making of buying/selling a stock is given by strategies which are reference tables [18]. An example of a strategy is shown in Table 1. A strategy tells what decision to take (either buy or sell) depending on the past price history of up (represented as “1”) and down (represented as “0”) price moves.

At each time step t agent i uses his/her optimal (i.e. best performing in terms of payoff, see definition below) strategy out of the s available to make an action 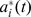 of either buying (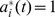) or selling (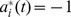) a stock. Notice that choosing the **optimal** strategy at each time step (indicated by the *) renders the model highly non-linear, since as the market changes the pool of optimal strategies also changes, which thereby in itself changes the market price behavior. The order imbalance at time t is given by
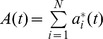
. The return, r(t), is assumed proportional to the order imbalance: 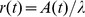
with λ being the liquidity. The payoff function 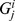
for the *j'th* strategy of an agent *i* is updated at each time step according to 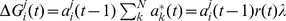. From the last equation one can see the reason for the name of the $G: assuming that a strategy recommended buying at time *t−1* (i.e. 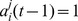), then it depends on the return in the following next time step, 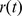, whether this recommendation turns out to be profitable or incurring a loss.

In short, the fitness of the $G strategies is determined by how well they predict which way the market will move one time step ahead. The idea is that strategies that are able to forecast the next market moves will thrive in terms of wealth and eventually, through evolution, become the ones that determine future market moves.

Without any constraints on the amount of money or the number of stocks available to the agents, the optimal state for all players is such that all agents cooperate and make the same decision (either buy or sell). This is a Nash equilibrium for the $G given by Keynes' “Beauty Contest,” where it becomes profitable for the agents to guess the actions of the other participants and mimic their decisions (for a derivation of this solution see Appendix S1).

In addition to decision making based on technical analysis, it would seem natural to include in the model decision making based on the future expectations of dividends, as it is done in the rational expectations approach. This can easily be added to the model by assigning strategies based on fundamental analysis [19]–[20]. However, the aim of the present study is to consider the dynamics of pure collectively created speculative behavior. In relation to financial markets, one can therefore think of our experiments as done in such a short time scale, that no new information, which could change future expectations of dividends or risk aversion appears. Therefore the fundamental price is constant throughout the experiments and equals the arbitrary price value chosen at time t = 0. The optimal state of the $G is the solution in which the price deviates exponentially in time from the fundamental value of the asset, since in this state all agents either profit from constant price increases by buying shares (this state we define as a “bubble”) or from constant price decreases by selling shares (this state we define as an anti-bubble). However, finding the optimal solution requires coordination between the agents to enter and stay in such states. Coordination is not intentional; rather, it emerges as a sum of independent decisions of agents choosing optimal strategies. These optimal strategies presented in the reference tables lead to the same action, which on an aggregate level means synchronization.

Strategies of the agents, at a first glance, are very different from what we know about decision heuristics of humans [21], [22], which are captured in terms of verbally (or rather propositionally [23]) formulated conditional rules. Clearly, humans are cognitively incapable of precisely representing the many vectors and exact sequences of market dynamics needed to represent and valuate the strategies. However, the reverse formalization of human decision heuristics by reference tables is simple and any conditional rule of human reasoning can be represented this way. To accept the notion that strategies of the agents represent human decision heuristics we need only to assume that each agent's strategy depicts in an algorithmic way the implementation of a decision heuristic that humans would specify in a higher-level language.

### Decoupling and synchronization

Before we present the experimental setup and results in more detail, we must first define the notion of decoupling. Some strategies represented by reference tables have a unique property: the actions that they recommend are decoupled from the incoming information. Let 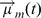 denote the price history of the last *m* up and down movements at time *t*. A strategy is called (1-time step) decoupled, if the action of the strategy at time *t*+*2*, 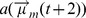, does **not** depend on 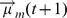. If 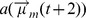 **does** depend on 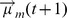 the strategy is called coupled to the price history [24].

Decoupling of a strategy means that different patterns of market history lead to the same decision (i.e. buy or sell), regardless of whether the market went up or down in the time step preceding the one in which the decision is to be made. The most interesting in the mechanism of decoupling is, as we will show, that it gives a handle to predict biased price behavior before it can be seen in the price data.

The strategy in Table 1 is one time step decoupled conditioned on the price history 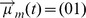 at time *t,* because independently of whether the market at time *t*+1 goes up ((01)→(11)) or down ((01)→(10)), the strategy will recommend buying at time *t*+2. This means that every time we see an occurrence of the price history where the market first went down (0), then up (1) we know for sure what action this strategy will recommend two time steps ahead. In a hypothetical game with only one agent and only one strategy, that presented in Table 1, we would know with certainty what the agent would do at time *t*+2 if the price history at time *t* was (01), **independently** of the price movement at time *t*+1.

So far we showed how an analysis of the agents' strategies could lead to momentary predictability of their future actions. But knowing for sure what one, or even several agents will do, does not guarantee being able to predict what will happen at the level of the market.

To know for sure how the market will behave, we need to encounter a situation in which not only are a majority of agents decoupled, but they need to be decoupled in the same direction. At any time *t*, the actions of agents can be thought of as coming from either coupled or decoupled strategies. The order imbalance can be written in terms of two distinct contributions: 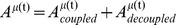. The condition for certain predictability of what will happen one time step ahead is therefore 
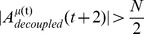
 because in that case we know that, given the price history at time *t*, the sign of the price movement at time *t*+2 will be determined by the sign of 
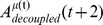
.

Whenever the condition
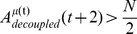
 is fulfilled we say that the system has synchronized. A priori, it is highly nontrivial whether this condition will be fulfilled at any point in time. If the agents play their strategies randomly, the condition is never fulfilled [24]. Decoupling therefore has to be related to the dynamics of pricing, which somehow imposes that the optimal strategies of agents will be attracted to regions in the phase space of strategies that have decoupled ones. In the $G, the two most trivial strategies with actions either all +1 or all −1 are natural candidates to be attractors. However, because it is very unlikely for an agent to possess either of these two strategies, an attractor would necessarily have to consist of regions in the phase space of strategies where one finds strategies highly similar to those that have all actions either +1 or −1.

In terms of the decision heuristics of humans, decoupling may be translated as a cognitive mechanism called cognitive closure which affects market players who stick to their long term decision regardless of what happens in the near future. After observing certain patterns of market dynamics, investors may come to the conclusion that the market trend is set and, further, that the temporary market reversals are not indicative of the real market trend. For example, if the market player judges that the market is trending upwards, then the increase in price serves as a confirmation of the expected trend, so the decision is to buy. If the price drops, it is perceived as a momentary deviation from the governing trend, which indicates immediate correction, so the decision is also to buy.

## Results

### Experimental design

Our experimental procedure is designed to investigate the emergence of collective speculative bias, and to demonstrate how agent-based simulations can be used to explain and predict it. We first performed multi-agent Monte Carlo simulations (i.e., no human decision making was involved) to study the theoretical aspects of speculation in the $G. Secondly we performed experiments with human subjects playing the $G, without the involvement of artificial agents (i.e., the experiments were done uniquely with human subjects). Finally we used data generated by humans as input for the multi-agent Monte Carlo simulations. In all three types of procedures (simulations, experiments on humans, simulations with data from experiments on humans), the rules of the $G were applied [17], and the parameters of the game were the following: (a) the number of players *N* = 11; (b) in the first trial the length of the memory m = 3, in the second trial m = 6 time steps; (c) the strategies will be described in each of the procedures separately.

### Multi-agent computer simulations

We first examined the intrinsic properties of the $G with respect to the creation of speculative biases. We focused on how often decoupling would be the responsible mechanism leading to an onset of bubbles/anti-bubbles. As shown in Appendix S1, the optimal solutions of the $G (without any constraints on the agents in terms of wealth/stock possessions) are either a bubble state, in which a majority of agents buys a new asset at each time step, or an anti-bubble state, in which a majority of agents short sells the assets, in both cases to the benefit of the majority. We therefore generated *L* = 50000 Monte Carlo (MC) simulations of the $G with fixed m and *N* corresponding to the two trials (*m* = 3, *N* = 11 and *m* = 6, *N* = 11). In every simulation, the total pool of the strategies *s* held by an agent was random (1<*s*<50, with *s* being drawn each time independently from a uniform distribution), and the sub-pools of strategies assigned to each of the agents was also randomly generated at the beginning of each game. Each of the MC simulations was then run until either a bubble or an anti-bubble was created (a bubble/antibubble being defined as *m* consecutive increases/decreases of the market).

For *m* = 3, we found that 57% of the bubbles/anti-bubbles entered a state of decoupling, whereas for *m* = 6 54% of the generated bubbles/anti-bubbles were in a state of decoupling. We can therefore claim that decoupling is a sufficient but not necessary condition for the occurrence of synchronization. To establish to what extent the decoupling of agents' strategies can act as an early predictor of bubbles or anti-bubbles, we performed the following numerical experiment. For each of the decoupled bubbles/anti-bubbles, we studied, as a function of time *t_b_* - *t*, the percentage 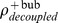 of decoupled optimal strategies used by agents at a given moment that recommend a decision along the direction of the bubble/anti-bubble, as well as the percentage 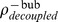 of decoupled optimal strategies with decisions in disagreement with the direction of the bubble/anti-bubble. The time of the onset of a bubble/anti-bubble, *t_b_*, is defined as the moment at which the price begins to constantly increase or decrease. The average value of *t_b_* depends only on the length of the memory, and we found that it can be scaled as 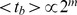.

**Figure 1:**
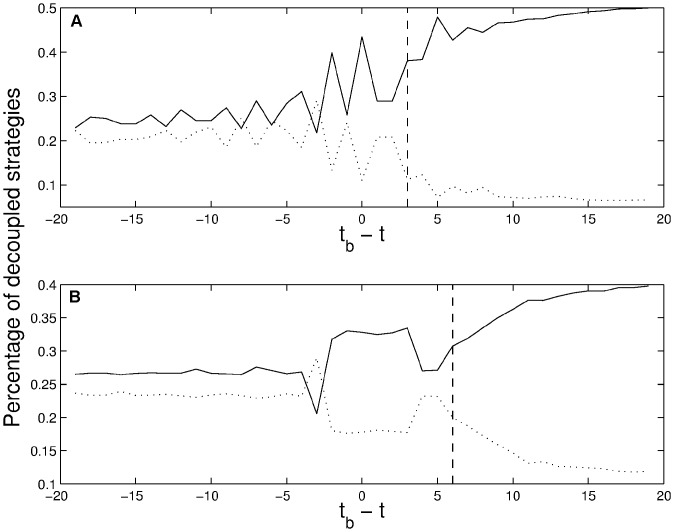
Splitting in the agents' use of different optimal decoupled strategies (solid and dotted lines) indicating an onset of a speculative bias in computer simulations of the $-game. The splitting as a function of time allows predicting the presence of a speculative bias before it can be seen in the price history generated by the agents. Solid line indicates the percentage of optimal decoupled strategies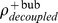 used by agents who at a given moment recommend a trading decision along the direction of the bubble/anti-bubble. Dotted line indicates the percentage 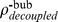
of decoupled optimal strategies with trading decisions in disagreement with the direction of the bubble/anti-bubble. Time is normalized in such a way that the moment when a speculative bias begins (defined as the moment at which the price begins to constantly increase or decrease) corresponds to t = t_b_. It is therefore not before t = t_b_+m (i.e., only after having observed m consecutive price increases/decreases) that a collective speculative bias can be defined ex post from the price time series. The vertical dashed lines indicates this moment for length of memory m = 3 (graph A) and m = 6 (graph B). The observation that a split between the solid and dotted lines occurs before the onset of a bubble (indicated by t_b_) means that prediction of biased price movements in the game is possible before they are actually visible in the prices.

The plots presented in Figure 1 show a splitting in the agents' use of the different optimal decoupled strategies (solid and dotted lines), which indicates an onset of speculative bias in computer simulations of the $-game. The splitting as a function of time allows to predict the presence of a speculative bias **before** it can be seen in the price history generated by the agents. It is remarkable that for *m* = 6 a clear split is observed as early as 20 time steps before *t_b_*; it should be noted that until *t_b_*+*m*−1, any predictability is nontrivial because only at time *t_b_*+*m* do the agents encounter *m* price changes in the same direction.

### Experiments with human subjects

In experiments with human subjects one of the main questions was whether the behavior of humans would be similar to that of the artificial agents, with respect to the spontaneous formation of speculative bias. Although the optimal solution in the $-Game is given by the formation of bubbles/anti-bubbles, we know that even in simple games people do not always find the Nash equilibrium. The experimental literature is full of examples of games in which the observed behavior quickly converges to equilibrium, as well as games in which equilibrium is a persistently poor predictor of humans' behavior [25]. In case of a successful reproduction of synchronization among human subjects playing the $G, we hypothesized that coordination between subjects, which triggers bubbles or anti-bubbles, is associated with the subjects' ignorance of the incoming information, corresponding to the agents' decoupled strategies.

The experiments were conducted on humanities students at the University of Warsaw. The experiment was conducted in eight groups, each composed of 11 participants. Before the participants logged into the game, they were informed about the details of the experiment with both verbal and written instructions, and asked to give informed consent. Participants were instructed to play the $G, i.e., to make buy/sell decisions according to their predictions of the future price movement (see the Method section and Appendix S2 for a detailed description of the procedure and instructions given to the subjects).

To test the hypothesis concerning the investors' loss of adaptability to incoming information (the sensitivity to the direction of price change during the bubble/anti-bubble state) we manipulated the direction of the price change at some point in the game. In four out of eight groups (nos. 5, 6, 7, and 8) the experimenter modified the sign of the price change to the opposite of the ‘real’ one derived from the players' decisions, i.e., introduced false feedback. The participants were not informed about this manipulation prior to the experiment. In two groups (nos. 5 and 7) false feedback was introduced once, and in the other two twice – every time after the subjects stayed for a longer time in a bubble/anti-bubble state (at least 15 time steps of consistent ‘ups’ or ‘downs’). The introduction of a false feedback meant that the subjects saw a response of the market opposite to what should have resulted from their actions - a minus instead of a plus or vice versa.

All of the experiments involving human subjects were conducted following approval of the procedure granted by the Research Ethics Committee of the Department of Psychology, University of Warsaw. To ensure anonymity of subjects informed consent was obtained from all subjects in oral form.

In all but one group (88%) the participants managed to synchronize, regardless of the length of the given history: six times they created a bubble, and once (no. 7) they created an anti-bubble. Only group no. 1 failed to create a bubble or an anti-bubble but they did manage to create short periods of synchronization, which could be the result of a ‘return to the mean’ strategy [26]. This strategy says: ‘when the price keeps increasing over (approx.) 5–6 time steps in a row, start selling, and when the price keeps decreasing over (approx.) 2–3 time steps in a row, start buying.’

Results of the experiments with human subjects confirm that in the $G subjects are capable of coordinating to achieve a market behavior that is most profitable for them, i.e., a monotonic series of either constant buying or selling. However, contrary to artificial agents they were also able to find other solutions than the pure bubble/anti-bubble state.

### Monte Carlo simulations input with data generated in experiments with humans

Having performed the trading experiments with human subjects, we used the price data generated in these experiments as input to agent-based Monte Carlo (MC) simulations of the $G. The MC simulations were performed with fixed m and *N* corresponding to the two trials (*m* = 3, *N* = 11 and *m* = 6, *N* = 11), a random number of strategies s, 1<s<50 (since a priori we do not know the number of strategies used by the human subjects) and their random initial realization (i.e. using a randomly generated string of 0 s and 1 s with an equal probability of 0.5, see right column in Table 1) of each of the s strategies. For each experiment the price data generated in experiments with humans was used as input price data used by the agents, and 50000 MC randomly generated realizations were then used to make an average estimate of 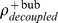 and 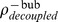.

**Figure 2:**
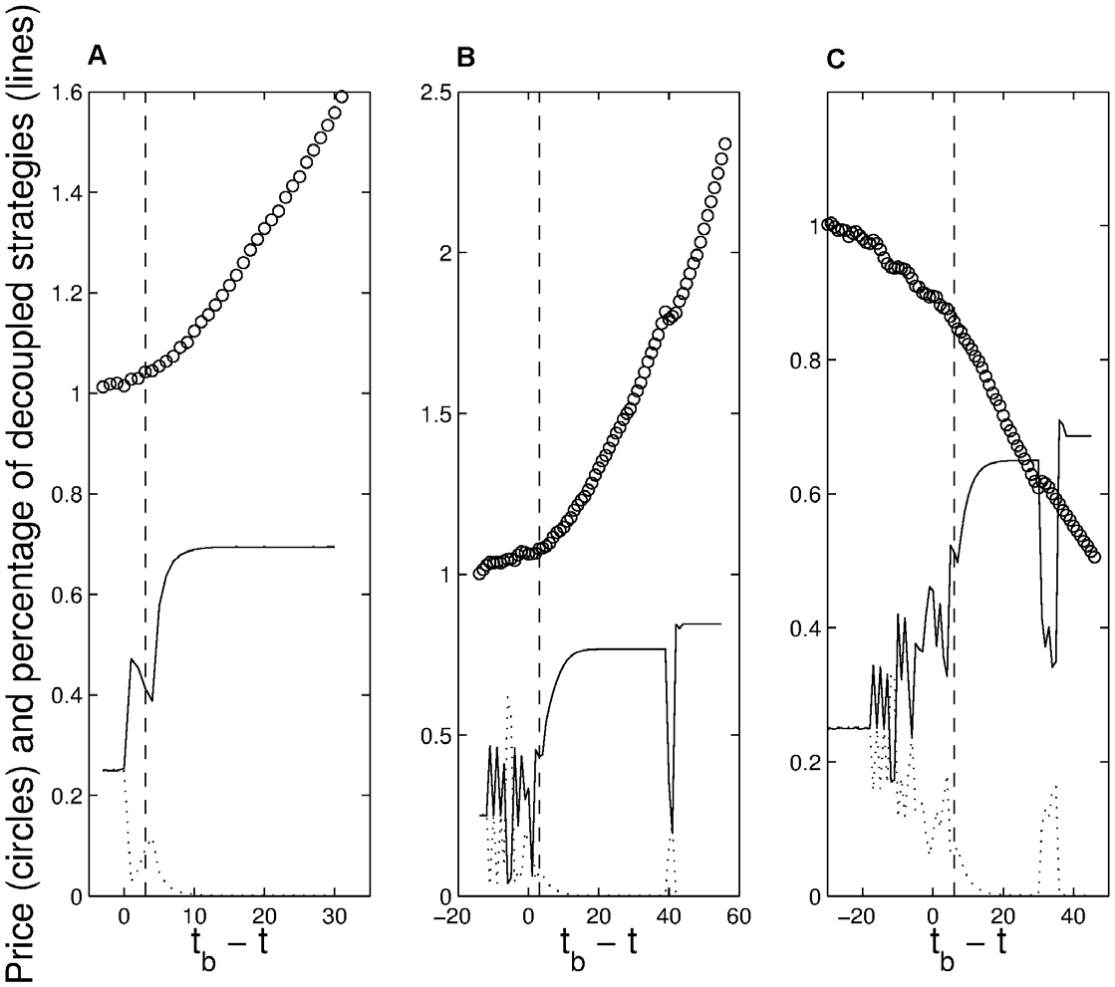
Speculative biases in the price movements - indicated by circles - in experiments with human subjects. The onset of the speculative bias was subsequently detected in Monte Carlo simulations with agents of the $-game trading on the price data generated by the humans. The rather sharp transition in the splitting of solid and dotted lines over time (for definitions of the different lines see caption 1) can be used to mark the onset of the speculative bias before it is visible in the price history. The lengths of memory used in experiments with human subjects were m = 3 (graph A and B) and m = 6 (graph C).

Figure 2 shows how the rather sharp transition in the splitting of the solid (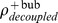) and dotted (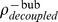) lines over time can be used to mark the onset of the speculative bias in the human trading experiments **before** it is seen in the generated price history. Even when a bubble is created very rapidly, we see a split (Figure 2A); this split becomes clearer over a longer time period for the larger memory length (*m* = 6), in which case the subjects have a longer period over which they trade in a descending market before the final synchronization occurs (Figure 2C). This resembles real markets, with a typical run-up/-down before a bubble/anti-bubble sets in.

We found that this method of detecting decoupled strategies of agents and predicting the synchronization of human subjects can be used to discover not only bubbles or anti-bubbles but also short moments of synchronization among the human subjects.

**Figure 3:**
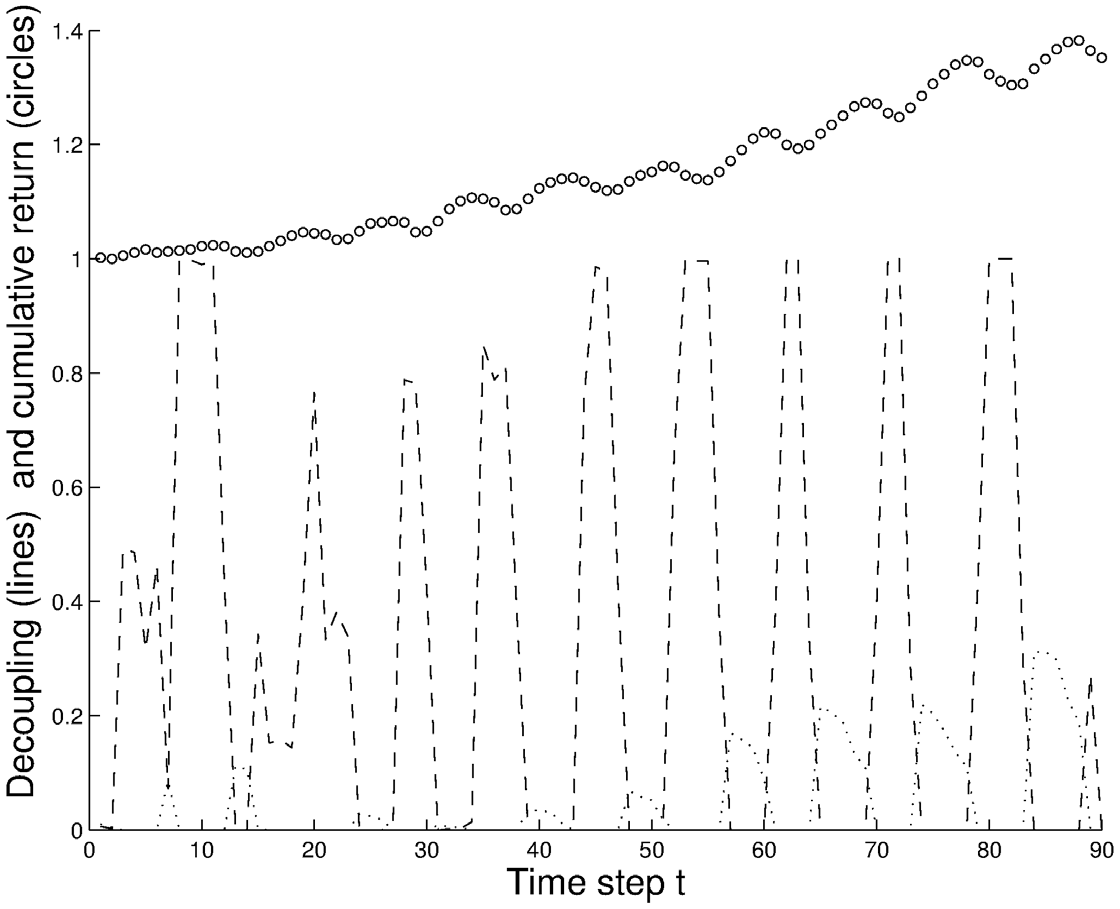
Illustration of a biased price evolution - indicated by circles - leading to “moments of predictability” in an experiment with human subjects (group no. 1, length of memory m = 3). This is the only experiment in which a clear bubble/anti-bubble was not created. The price data generated in these experiments were used as input to agent-based Monte Carlo (MC) simulations of the $G. Dashed lines indicate the percentage of optimal strategies in the Monte Carlo simulation decoupled along the direction of the price increase, whereas the dotted lines indicate the percentage of optimal strategies against the direction of the price increase. The clear “peaks” in the dashed lines predict a price increase before it can be seen in the experiment.

Figure 3 shows the course of the one experiment in which human subjects did not generate a clear bubble or anti-bubble (group no. 1). Still by applying the same MC analysis as in Figure 2, clear “peaks” in the value of 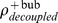 predict a price increase **before** it is actually seen in the experiment. We found a stunning 87% success rate of predicting a single move of the market two time steps in advance. It is important to note that there are no parameters used in the predictions, which were made out-of-sample.

Finally we wanted to verify if decoupling corresponds to moments of collective bias where the human subjects collectively neglect incoming information, so we focused on the four experiments, in which we manipulated the price change after a clear price trend leading to a bubble/anti-bubble had been established (introduction of false feedback). In two groups (5 and 7) detecting decoupling among a majority of MC agents (75% and 65%) in the time step preceding the introduction of the false feedback indicated that humans are in a state of decoupling, which predicted that they would not react to a disconfirming piece of information. Indeed, the humans did not react to the price manipulations in these two cases. In the other two experiments (groups no. 6 and 8), the low level of decoupling found in the MC simulations at those moments made predicting human reactions to false feedback impossible.

## Discussion

We have performed trading experiments with human subjects to study the dynamics of collective decision making in a simple model of financial markets. The experiments show how certain spontaneous collective “moods” or “biases” can emerge dynamically. Introducing a theoretical framework and applying computer simulations to the data generated by the humans, we have been able to detect the onset of such moments **before** they actually happen in the experiments. Our experiments and theoretical framework suggest new ways to access the pathways involved in collective formation of speculative behavior.

## Methods

### Ethics Statement

All of the experiments involving human subjects were conducted following approval of the procedures (including oral informed consent procedure) granted by the Research Ethics Committee of the Department of Psychology, University of Warsaw. We chose the oral informed consent procedure to ensure anonymity of participants and because the research presents no more than minimal risk of harm to the subjects and involves no procedures for which written consent is normally required outside of the research context. In order to obtain informed consent, the following steps were conducted. First the experimenter explained the procedure of the study to the potential subject verbally, providing all important information: aim, rules of the game, remuneration, duration. Then the potential participants were given written instruction containing the description of the game and their role in it, as well as time to ask any questions. Only then the decisions whether or not to participate in the experiment were made. The procedure of collecting informed consent was witnessed by at least 10 other potential participants. Participants who gave informed consent verbally were instructed to log into the game. Each participant was assigned number from 1 to 11 according to the order they entered the game (numbers were not revealed to participants).

### Detailed description of the procedure in the experiments with human subjects

The experiments with human subjects were conducted in one of the computer laboratories at the University of Warsaw. The subjects were students in humanities who responded to an announcement about the study, or found out about it elsewhere (e.g. from other participants) and expressed their willingness to participate. Each experiment required the participation of 11 subjects, so altogether we gathered 88 participants (forming 8 groups), all of which declared a lack of experience in investing in stock exchanges and no prior economic education.

The participants were first informed about the details of the experiment and expressed informed consent to participate. Then they were given instructions concerning the $G. They were told that in each time step, their behavior influences the price. They had no time limit for making their decisions, but they knew that at every time step the new price would be set only after everyone (all 11 subjects) had indicated their decisions (buy or sell). At each time step, the price was the result of the subjects' decisions and it was set according to: return = (order imbalance)/liquidity, where the order imbalance was the number of participants that bought a share minus the number of participants who sold a share. Liquidity was taken constant. At the beginning of the game, participants had no information about the market or the price on the market. During the game, participants had access to only four pieces of information (same as artificial agents in MC simulations): (a) m last price changes indicated by sequences of “+” and “−” (in the first trial m = 3, in the second trial m = 6); (b) own last decision, indicated by a “+” or “−”; (c), own profit from the last decision; and (d) own cumulative profit from the whole game. Each of the experiments lasted up to an hour. Having finished the game, the participants were remunerated for their participation. They received 5 Polish Zlotys (PLN), which is the equivalent of approximately $2, show-up fee and on top of that they were given 1% of their virtual payoff from the game. This way the subject could receive around 15pln altogether (approximately $6).

## Supporting Information

Appendix S1 **Optimal solution of the $-Game.**
(DOC)

Appendix S2 **Instructions given to the subjects in experiments on humans (history m = 3).**
(DOC)

Figure S1 **Screenshot of a representative game in time step t = 1.**
(TIF)

Figure S2 **Screenshot of a representative game in time step t = 2.**
(TIF)

Figure S3 **Screenshot of a representative game in time step t = 3.**
(TIF)

Figure S4 **Screenshot of a representative game in time step t = 4.**
(TIF)

Figure S5 **Screenshot of a representative game in time step t = 5.**
(TIF)

Figure S6 **Screenshot of a representative game in time step** ***t*** **= 6.**
(TIF)
