# Supplementary material for: Short and Long Term Investor Synchronization Caused by Decoupling
Source: PLoS One. 2012 Dec 7;7(12):e50700. doi: 10.1371/journal.pone.0050700 (PMC3517516; doi:10.1371/journal.pone.0050700)
Supplement: Appendix S1 — Optimal solution of the $-Game. (DOC) [file pone.0050700.s001.doc]

**Optimal Solution of the $-Game S1**

We will first introduce the Minority Game in terms of a general dynamic equation and will then at the end of the appendix show the specific solution within such a framework for the Dollar Game.

In the Minority Game (MG), *N* agents possess strategies *S_i_(t)* that use the direction of the last *m* price as a binary string, $\mathop{\text{µ}}\limits^{\to} (t)$, of zeroes (downward movement of the market) and ones (upward movement of the market) to choose one of two alternatives (buy or sell a share) at time *t*. Each agent is initially assigned *s* (in general) different strategies. At each time step *t* an agent uses his/her current best (indicated by a star) performing strategy so far to take the action $a_{i}^{*}=\pm1$ of either buying $a_{i}^{*}=1$ or selling $a_{i}^{*}=-1$ a share. The optimal strategy of the agent is determined by the payoff function $G_{i}^{MG}$ updated at every time step according to $\delta G_{i}^{MG}=-a_{i}(t)A(t)$. The sign of A(t) in turn determines the value of the last term b(t) in $\mathop{\text{µ}}\limits^{\to} (t+1)$ with $A\text{(µ}(t))=\sum_{i=1}^{N} a_{i}^{*}\text{(µ}(t))$. Instead of the usual algorithm describing the dynamics of the MG, it can then be summarized into one equation:

 $b(t+1)=\theta(A(t))=\theta(\sum_{i=1}^{N} a_{i}^{*}\text{(µ}(t)))$ , *(A1)*

here θ is the Heaviside function and $\text{µ}(t)=\sum_{j=1}^{m} b(t-j+1)2^{j-1}$ is now represented now as a scalar. As can be seen from (A1) the price setting is determined by the order imbalance A(t) with a positive imbalance giving rise to a price increase and a negative order imbalance giving rise to a price decrease. The results obtained in the following are therefore very general and any functional form for price setting obeying this simple and natural condition, will lead to the same Nash equilibriums found below.

$a_{i}^{*}\text{(µ}(t))=S_{i}^{\{j\left|\max_{j=1,...,s}\{G(S_{i}^{j}\text{(µ}(t)))\}\right.}\text{(µ}(t))$, and

$G^{MG}(S_{i}^{j}\text{(µ}(t)))=\sum_{k=0}^{t} -S_{i}^{j}(\text{µ}(k))A(\text{µ}(k))$ (A2)

With $A(\text{µ}(t))=\sum_{i=1}^{N} a_{i}^{*}(\text{µ}(t))$. Inserting the expressions (A2) in the expression for *b*(*t*) (A1) one gets an expression that describes the Minority Game in terms of just one single equation for *b*(*t*) depending on the values of the variables *m, s, N* and the quenched random variables $S_{i}^{j}$. A major complication in the study of this equation occurs because of the nonlinearity in the selection of the best strategy. For *s* = 2 however the expressions simplifies because one only needs to know the relative payoff $q_{i}\equiv G(S_{i}^{1})-G(S_{i}^{2})$ between the two strategies [1,2]. The action of the optimal strategy, $a_{i}^{*}$ can be expressed in terms of so that *A*(*t*) for *s* = 2 takes the form:

$$A(\text{µ}(t))=\sum_{i=1}^{N} a_{i}^{*}(\text{µ}(t))$$

$=\sum_{i=1}^{N} [\theta(q_{i}(\text{µ}(t)))S_{i}^{2}\text{(µ}(t))+(1-\theta(q_{i}\text{(µ}(t)))S_{i}^{1}\text{(µ}(t))]$ (A3)

$\frac{db}{dt}\left|t+1=\delta(\right.A(\text{µ}(t)))\frac{\delta A}{\delta t}$ (A4)

$$\begin{matrix} =\delta(A(\text{µ}(t)))\sum_{i=1}^{N} \{\delta(q_{i}(\text{µ}(t))\frac{\delta q_{i}(\text{µ}(t))}{\delta t}[S_{i}^{2}\text{(µ}(t))-S_{i}^{1}\text{(µ}(t))] \\ +\theta(q_{i}\text{(µ}(t))\frac{\delta S_{i}^{2}(\text{µ}(t))}{\delta t}+[1-\theta(q_{i}\text{(µ}(t))]\frac{\delta S_{i}^{1}(\text{µ}(t))}{\delta t}\} \end{matrix}$$

(A5)

From the bracket of the sum in (A5) a change of *A*(*t*) can arise either because the optimal strategy changes *and* the two strategies for a given , differ (first term in the bracket). Or *A*(*t*) can change simply because the optimal strategy changes its prediction for the given (second and third terms in the bracket). For the Minority Game and the $-Game, the relative payoff changes in time respectively as:

$\frac{\delta q_{i}^{MG}}{\delta t}\left|t=\right.-S_{i}^{2}\text{(µ}(t-1))A\text{(µ}(t-1))+S_{i}^{1}\text{(µ}(t-1))A\text{(µ}(t-1))$, (A6)

$\frac{\delta q_{i}^{\$G}}{\delta t}\left|t=\right.S_{i}^{2}\text{(µ}(t-2))A\text{(µ}(t-1))-S_{i}^{1}\text{(µ}(t-2))A\text{(µ}(t-1))$. (A7)

Inserting *μ* from eq. (A1) and inserting eq. (A7) in eq. (A5) one gets for the MG:

(A8)

One can make the analog for *b* as a ‘magnetism’ determined by the ‘spins’ represented by the strategies *S_i_*. The first term then correspond to the ‘interacting’ case stemming directly from the introduction of the payoff function with interaction between different spins (from products of *A* and *S_i_*). The second and third terms are ‘free field’ terms, the only terms present without a payoff function. In the case of the $G (A7) and with *A*(*μ*(*t-*1))*,A*(*μ*(*t-*2))*,…,A*(*μ*(*t-m*)) all having same sign, the r.h.s. of (A8) becomes 0, which shows that a constant bit, corresponding to either an exponential increase or decrease in price, is a Nash equilibrium for the $G.

**Supporting References**

1. Challet D, Marsili M (1999) Phase transition and symmetry breaking in the minority game. Physical Review E 60: R6271-R6274.

2. Marsili M, Challet D (2001) Continuum time limit and stationary states of the minority game. Physical Review E 64: 056138.
