# Supplementary material for: Short and Long Term Investor Synchronization Caused by Decoupling
Source: PLoS One. 2012 Dec 7;7(12):e50700. doi: 10.1371/journal.pone.0050700 (PMC3517516; doi:10.1371/journal.pone.0050700)
Supplement: Appendix S2 — Instructions given to the subjects in experiments on humans (history m = 3). (DOC) [file pone.0050700.s002.doc]

**Instructions given to the subjects in experiments on humans (history m=3)**

Imagine that this is the end of the summer. Farmers began to harvest turnip, while vegetable processing plants are preparing for the opening of the season. The beginning of the game is an official opening of the trade. The official price of turnip is set daily, and only at that price you can sell or buy a turnip. Price is established each morning on the basis of supply and demand - if there is more people willing to buy turnip than sell, the price is rising and vice versa). The size of price change depends on the size of the difference between supply and demand.

As it usually happens, farmers are not able to get along with the processing plants, therefore key players are the middlemen.

Your task is to put yourself in the role of a middleman.

The middleman has two options of earning from the transaction:
1. They may first make a deal with the farmer, i.e., they buy turnips from the farmer and then go to the processing plant in the consecutive time step and sell them commodity
2. They may first make a deal with the processing plant, to which they sell turnips and only then they go to the farmers to buy and provide turnip to the plant.

The decision to buy (sell) turnip at one day automatically results in the sale (purchase) of turnip at the next day.

It takes one day to move between the farmer and the factory, which means that when you buy turnips from a farmer at day *t* at the price of that day, you sell it to the processing plant the next day *t +1* at the market price at day *t+1*. Likewise, when you first sell turnip to the proceeding plant at day t at the price of that day, you buy it from the farmer the next day *t* *+1* at the market price at day *t+1.*

The choice of the option depends on the anticipation of price change. If the middleman predicts that a price will increase he should first make a deal with the farmer; buy turnip at the current price and then go to the processing plant and sell turnip at a higher price. If the predictions are confirmed, the middleman will earn from the difference between the buying price and selling price. If, on the other hand, the middleman’s prediction will prove to be wrong they buy a turnip at a higher price than the selling price.

If the middleman predicts the price drop they should first go to the processing plant and sell turnip at the prevailing price and then buy it from the farmers and provide it to the plant. If price actually drops a middleman earns, because they managed to sell turnips at a higher price than they bought it.

Remember that the official price bases on the demand – supply imbalance, and it is influenced by the decision of all middlemen (i.e., among others yours).

Eleven players take part in the game.

You will earn 5 PLN from taking part in the experiment and, in addition, you will be given the proportion of your virtual payoff from the game. Your virtual earnings will be divided by 100. The maximum you can earn is 15 PLN all together.

Details of the game are presented on the slides.

**Slide no. 1**

<Figure S1>

**Slide no. 2**

The beginning of the game

The beginning of the game is an official opening of the trade. Price is established each morning.

In the first time step, you need to decide whether you predict the increase of the price meaning that you go to the farmer to buy the turnip first (+) and then you go to the plant to sell it at a higher price or you predict decrease of the price so you go to the plant first and then you buy the turnip cheaper from the farmer (-). While making a prediction please indicate + or – in the box and click „NEXT>>” (Figure S1).

After clicking „NEXT>>” the graphical representation of your decision will be moved lower to the box (Figure S2).

The price will be set when all players will make their decisions in a given time step.

**Slide no. 3**

<Figure S2>

**Slide no. 4**

A representative screenshot during the second time step

The Figure S2 illustrates the situation in which a player started the game with the prediction of price decrease. The players do not know yet whether they earned or lost because the price change is not set until the next time step.

In the second time step, you have to make your second prediction. After pressing “Next>>” graphical representation of your decision will be moved lower on the screen and you will be shown the direction of the first price change (Figure S3).

**Slide no. 5**

<Figure S3>

**Slide no. 6**

A representative screenshot after the second time step

After the second step, the direction of the first price change and the change in cumulative payoff will be shown. Depending on the accuracy of your first forecast the payoff can be positive or negative.

The Figure S3 illustrates the situation in which a player in the first and the second time step predicted decrease of price.

The cumulative payoff of the player increased by 10 virtual units because they made a correct prediction in the first time step.

The value of the payoff depends on the magnitude of difference between the number of sellers and buyers on the market in a given time step.

**Slide no. 7**

<Figure S4>

**Slide no. 8.**

A representative screenshot after the third time step

After the third time step, the second price change will be shown.

The Figure S4 illustrates the situation in which a player predicted the decrease of price in the second and third time step. Because they made a correct prediction in the second time step they earned 30 virtual units.

Their cumulative payoff increased to 40 virtual units.

**Slide no. 9.**

<Figure S5>

**Slide no. 10**

A representative screenshot after the fourth time step

After the fourth time step, the third price change will be shown.

The Figure S5 illustrates the situation in which a player predicted increase of the price in fourth time step and decrease of price in third time step. Because they made a wrong prediction in the third time step (the price increased) their payoff decreased by 30 virtual units.

After the fourth time step the cumulative payoff of the player would account for 10 virtual units.

**Slide no. 11**

<Figure S6>

**Slide no. 12**

A representative screenshot after the fifth time step

After the fifth time step, the fourth price change appears and the first price change disappears from the screen (Figure S6). During the whole game you can track only three recent price changes.
