# Supplementary figures and images for: Short and Long Term Investor Synchronization Caused by Decoupling
Source: PLoS One. 2012 Dec 7;7(12):e50700. doi: 10.1371/journal.pone.0050700 (PMC3517516; doi:10.1371/journal.pone.0050700)

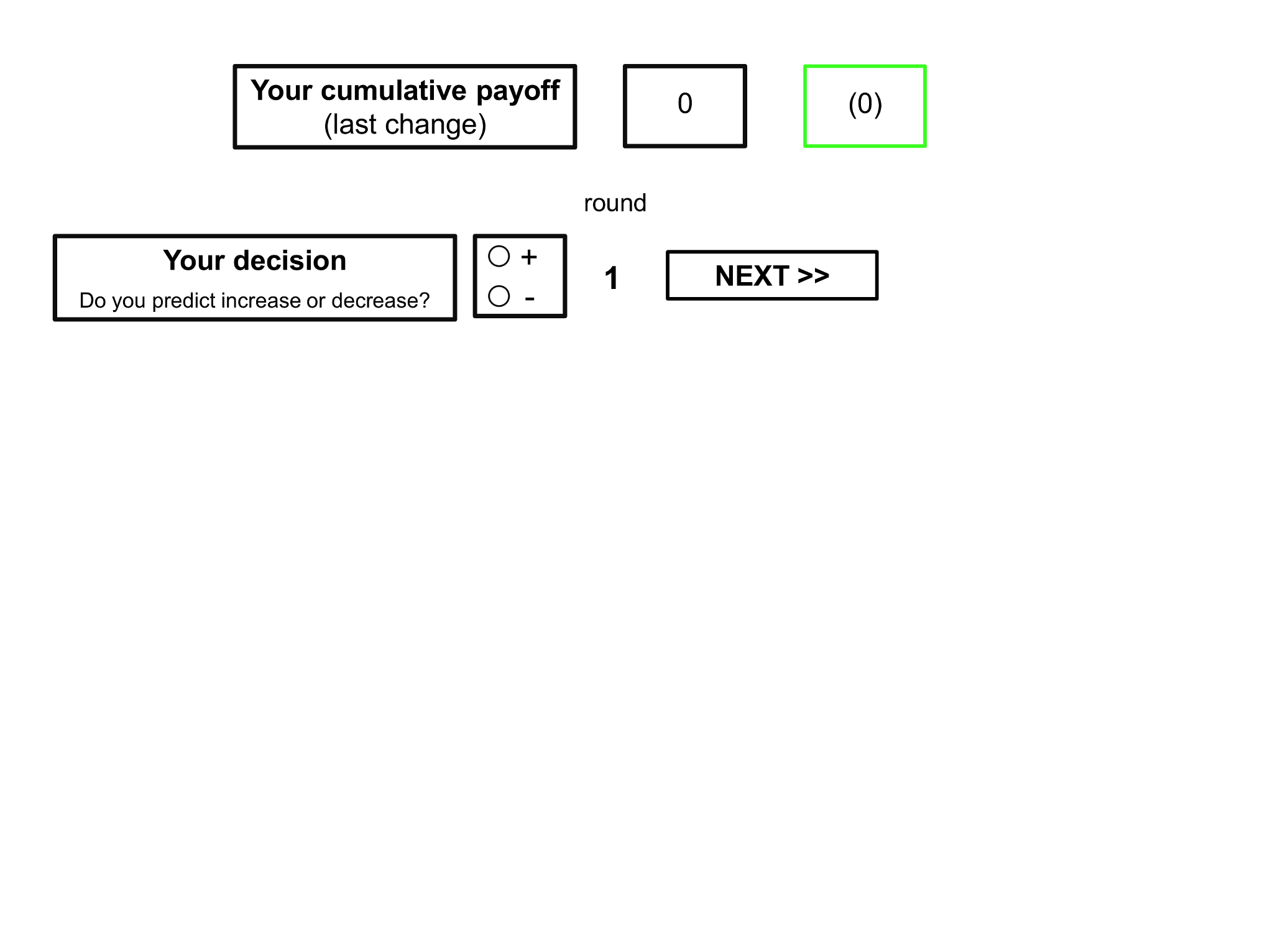

Supplement: Figure S1 — Screenshot of a representative game in time step t = 1. (TIF) [file pone.0050700.s003.tif]

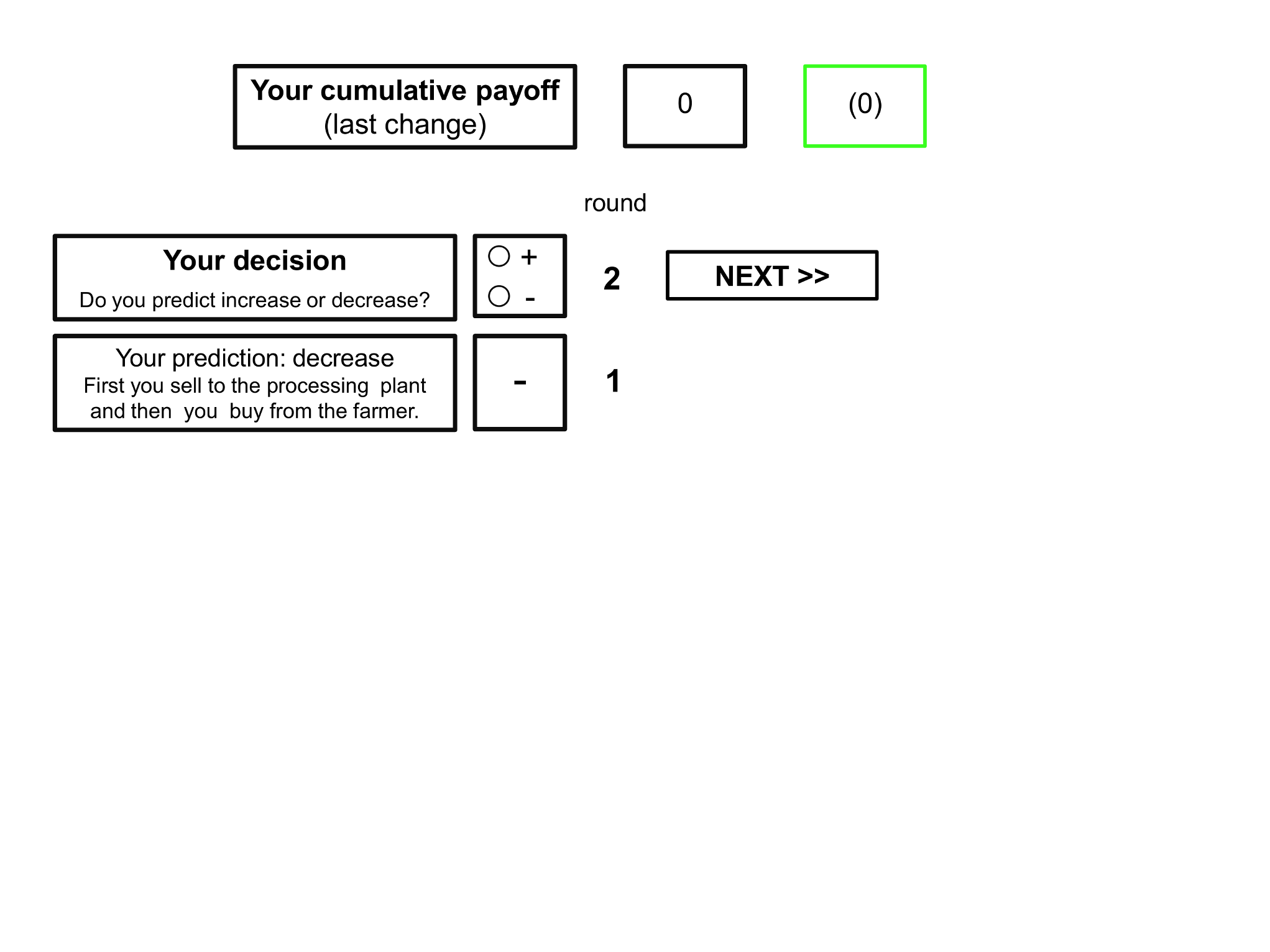

Supplement: Figure S2 — Screenshot of a representative game in time step t = 2. (TIF) [file pone.0050700.s004.tif]

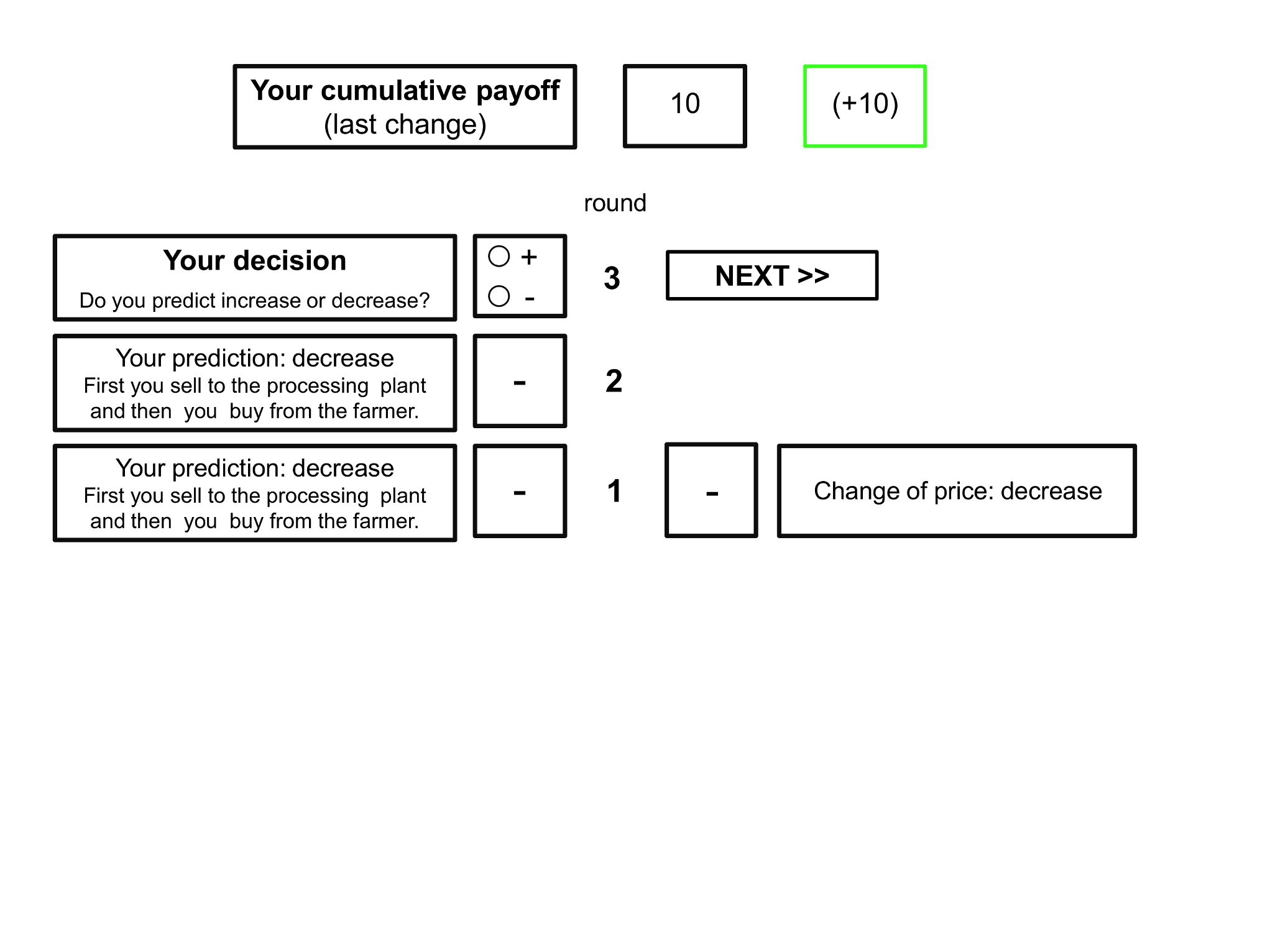

Supplement: Figure S3 — Screenshot of a representative game in time step t = 3. (TIF) [file pone.0050700.s005.tif]

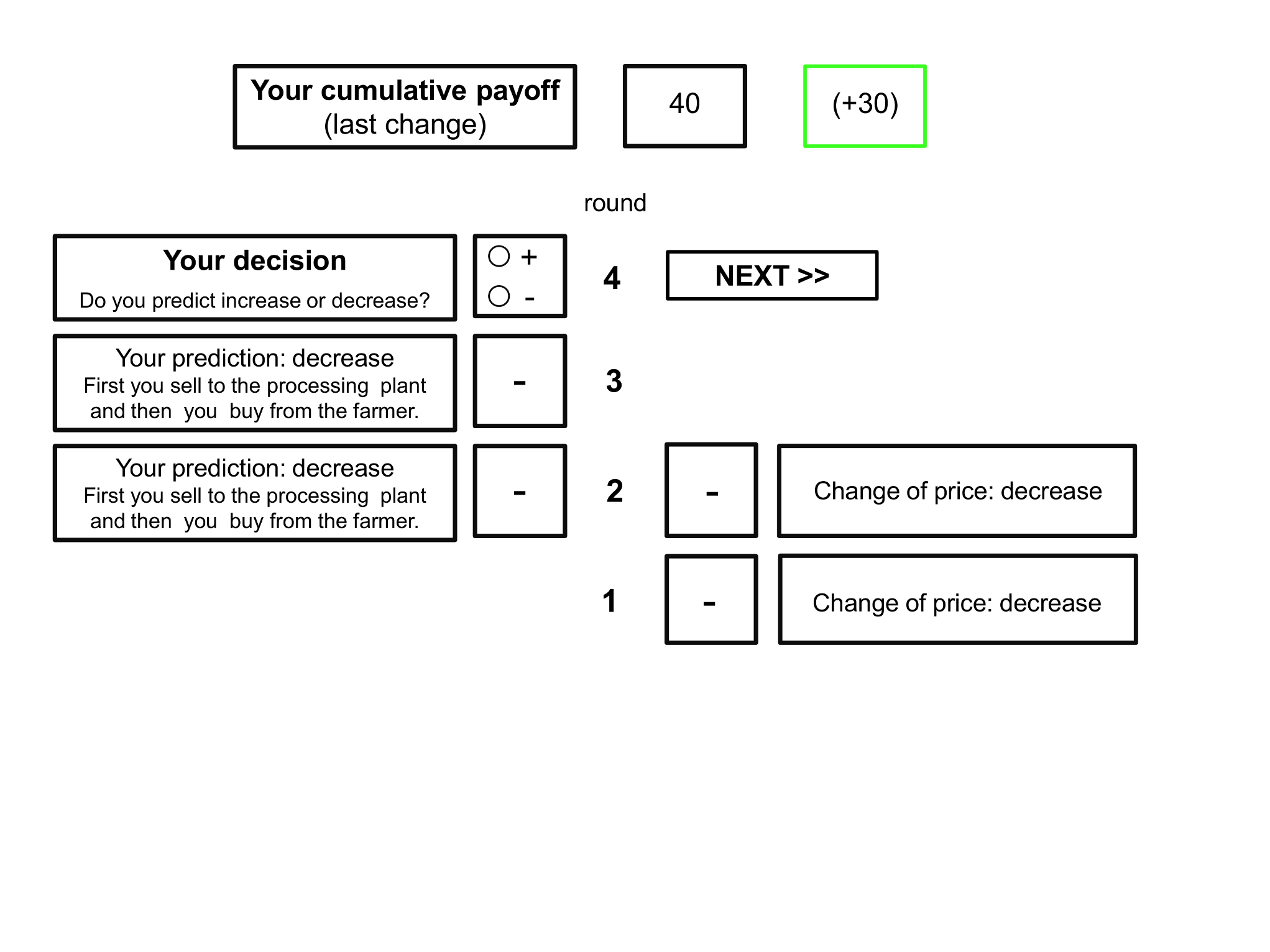

Supplement: Figure S4 — Screenshot of a representative game in time step t = 4. (TIF) [file pone.0050700.s006.tif]

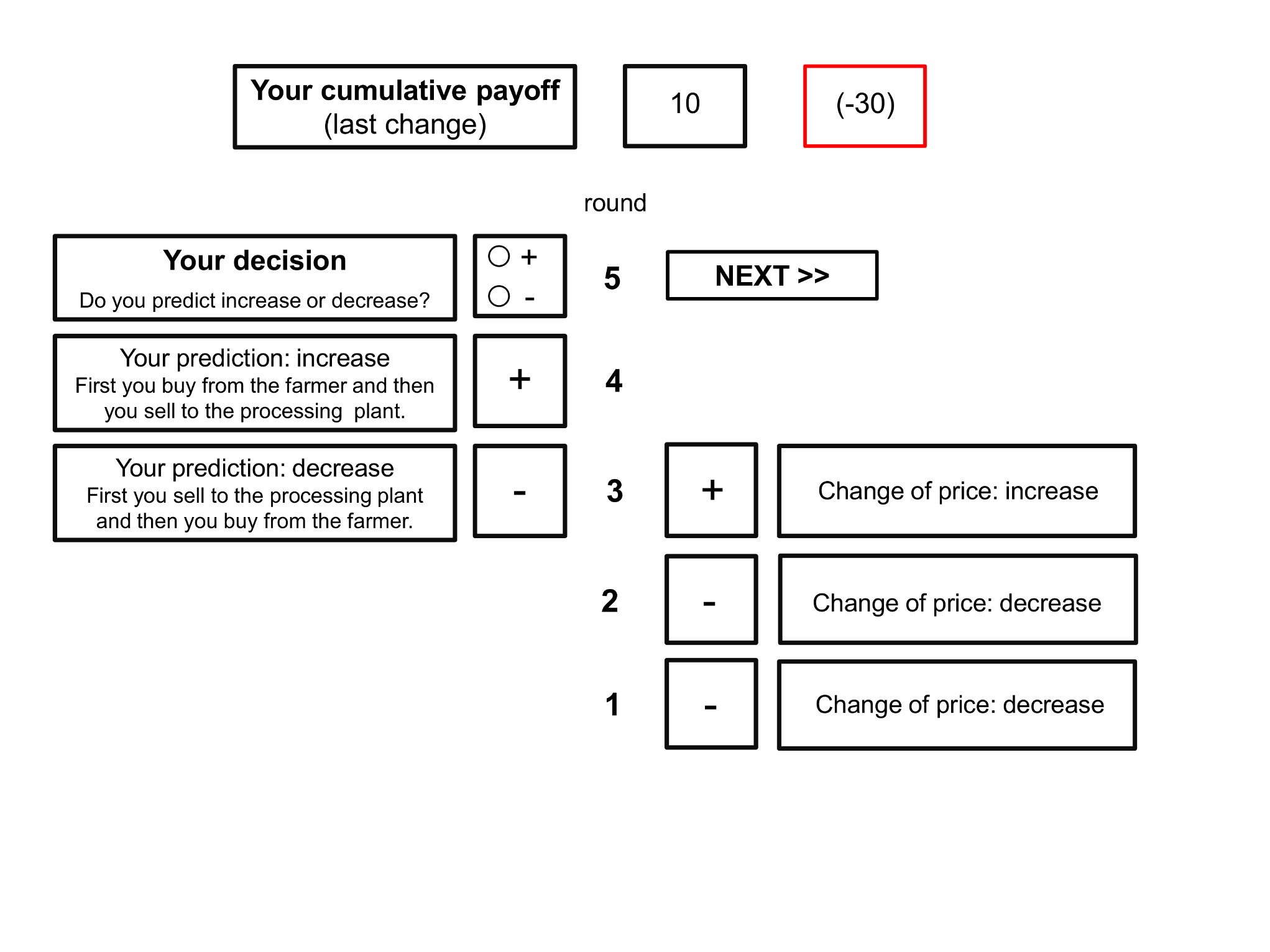

Supplement: Figure S5 — Screenshot of a representative game in time step t = 5. (TIF) [file pone.0050700.s007.tif]

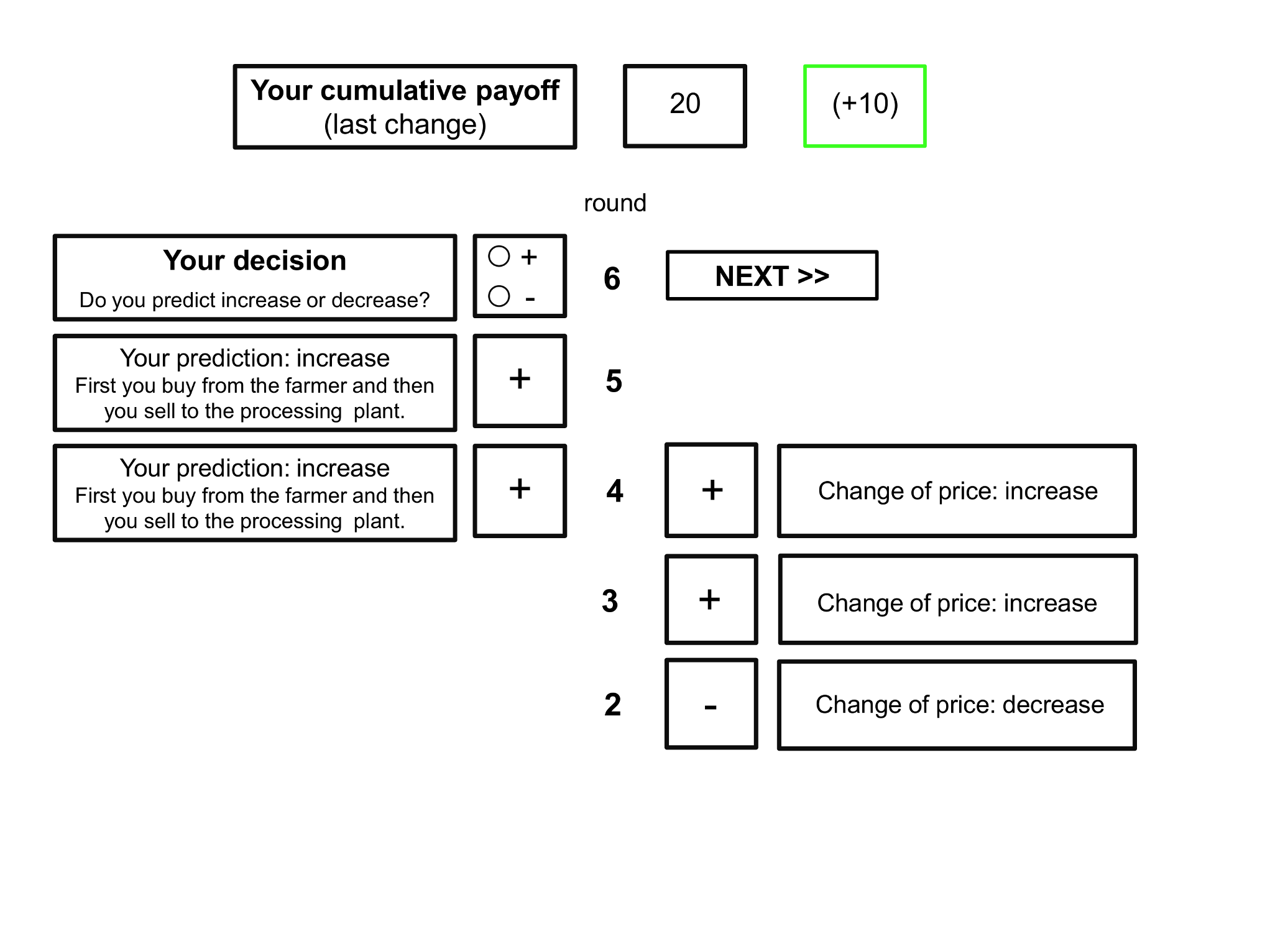

Supplement: Figure S6 — Screenshot of a representative game in time step t = 6. (TIF) [file pone.0050700.s008.tif]
